# Cerebral venous sinus thrombosis due to vaccine-induced immune thrombotic thrombocytopenia in middle-income countries

**DOI:** 10.1177/17474930231182901

**Published:** 2023-06-30

**Authors:** Anita van de Munckhof, Afshin Borhani-Haghighi, Sanjith Aaron, Katarzyna Krzywicka, Mayte Sánchez van Kammen, Charlotte Cordonnier, Timothy J Kleinig, Thalia S Field, Sven Poli, Robin Lemmens, Adrian Scutelnic, Erik Lindgren, Jiangang Duan, Yıldız Arslan, Eric CM van Gorp, Johanna A Kremer Hovinga, Albrecht Günther, Katarina Jood, Turgut Tatlisumak, Jukka Putaala, Mirjam R Heldner, Marcel Arnold, Diana Aguiar de Sousa, Mohammad Wasay, Antonio Arauz, Adriana Bastos Conforto, José M Ferro, Jonathan M Coutinho

**Affiliations:** 1 Amsterdam University Medical Centers, location University of Amsterdam, Amsterdam, The Netherlands; 2 Shiraz University of Medical Sciences, Shiraz, Iran; 3 Christian Medical College Hospital, Vellore, India; 4 Univ. Lille, Inserm, CHU Lille, U1172—LilNCog—Lille Neuroscience & Cognition, Lille, France; 5 Royal Adelaide Hospital, Adelaide, SA, Australia; 6 University of British Columbia, Vancouver, BC, Canada; 7 University Hospital Tuebingen, Eberhard-Karls University, Tuebingen, Germany; 8 University Hospitals Leuven, Leuven, Belgium; 9 Inselspital, Bern University Hospital, University of Bern, Bern, Switzerland; 10 Sahlgrenska University Hospital, Gothenburg, Sweden; 11 Institute of Neuroscience and Physiology, Sahlgrenska Academy at University of Gothenburg, Gothenburg, Sweden; 12 Xuanwu Hospital, Capital Medical University, Beijing, China; 13 Medicana İzmir International Hospital, Izmir, Turkey; 14 Erasmus Medical Center Rotterdam, Rotterdam, The Netherlands; 15 Jena University Hospital, Jena, Germany; 16 Helsinki University Hospital and University of Helsinki, Helsinki, Finland; 17 Lisbon Central University Hospital Centre, Lisbon, Portugal; 18 Aga Khan University, Karachi, Pakistan; 19 National Institute of Neurology and Neurosurgery, Mexico City, Mexico; 20 Hospital das Clínicas/São Paulo University, São Paulo, SP, Brazil; 21 Instituto de Medicina Molecular João Lobo Antunes, Faculdade de Medicina, Universidade de Lisboa, Lisboa, Portugal

**Keywords:** CVST, VITT, COVID-19, vaccination, global health, thrombosis

## Abstract

**Background:**

Adenovirus-based COVID-19 vaccines are extensively used in low- and middle-income countries (LMICs). Remarkably, cases of cerebral venous sinus thrombosis due to vaccine-induced immune thrombotic thrombocytopenia (CVST-VITT) have rarely been reported from LMICs.

**Aims:**

We studied the frequency, manifestations, treatment, and outcomes of CVST-VITT in LMICs.

**Methods:**

We report data from an international registry on CVST after COVID-19 vaccination. VITT was classified according to the Pavord criteria. We compared CVST-VITT cases from LMICs to cases from high-income countries (HICs).

**Results:**

Until August 2022, 228 CVST cases were reported, of which 63 were from LMICs (all middle-income countries [MICs]: Brazil, China, India, Iran, Mexico, Pakistan, Turkey). Of these 63, 32 (51%) met the VITT criteria, compared to 103 of 165 (62%) from HICs. Only 5 of the 32 (16%) CVST-VITT cases from MICs had definite VITT, mostly because anti-platelet factor 4 antibodies were often not tested. The median age was 26 (interquartile range [IQR] 20–37) versus 47 (IQR 32–58) years, and the proportion of women was 25 of 32 (78%) versus 77 of 103 (75%) in MICs versus HICs, respectively. Patients from MICs were diagnosed later than patients from HICs (1/32 [3%] vs. 65/103 [63%] diagnosed before May 2021). Clinical manifestations, including intracranial hemorrhage, were largely similar as was intravenous immunoglobulin use. In-hospital mortality was lower in MICs (7/31 [23%, 95% confidence interval (CI) 11–40]) than in HICs (44/102 [43%, 95% CI 34–53], *p* = 0.039).

**Conclusions:**

The number of CVST-VITT cases reported from LMICs was small despite the widespread use of adenoviral vaccines. Clinical manifestations and treatment of CVST-VITT cases were largely similar in MICs and HICs, while mortality was lower in patients from MICs.

## Introduction

Reports of thrombosis at unusual sites, especially cerebral venous sinus thrombosis (CVST), caused by the adenovirus-based COVID-19 vaccines ChAdOx1 nCov-19 (AZD1222; Oxford–AstraZeneca) and Ad26.COV2.S (Janssen–Johnson & Johnson) caused a major public concern in 2021.^1–3^ Although CVST due to vaccine-induced immune thrombotic thrombocytopenia (CVST-VITT)—as this disease is nowadays known—is rare, it is a severe condition that often afflicts young and previously healthy people.^2,3^

Almost all reports on VITT originated from high-income countries (HICs), which was initially not surprising, as these countries had preferential access to COVID-19 vaccines.^4^ Meanwhile, low- and middle-income countries (LMICs) had a delayed start to their vaccination campaigns.^5^ In response to the VITT occurrences, most HICs restricted, or outright banned, the use of adenovirus-based vaccines, relying on mRNA vaccines instead.^6^ In stark contrast, adenovirus-based vaccines remain vital for the vaccination campaigns in LMICs, as these vaccines generally are cheaper and easier to transfer and store.^7^ By 22 September 2022, 530 million doses of adenovirus-based COVID-19 vaccines have been secured through COVAX, which is a global collaboration for equitable access to COVID-19 vaccines.^8^ Moreover, in India alone, 1.67 billion ChAdOx1 nCoV-19 vaccines were administered by 23 August 2022.^9^

Vaccination campaigns in LMICs have accelerated in the past months and are still in full swing.^5,10^ However, despite the widespread use of adenovirus-based COVID-19 vaccines for millions of individuals in these countries, there are almost no reports on VITT from LMICs.^11,12^ A PubMed search on 15 December 2022 identified only nine CVST-VITT cases reported from LMICs (Supplemental Table 1). This apparent contradiction may be due to under-recognition of the condition in LMICs, for instance, due to limited availability of diagnostic tests such as vascular neuroimaging, or because some LMICs have a more nascent infrastructure for pharmacovigilance.^13^ Alternatively, or in combination with under-ascertainment, it may be that susceptibility to CVST-VITT differs across populations, as has, for instance, also been observed for the association between the Pandemrix H1 N1 vaccine and narcolepsy.^14^

The treatment of CVST-VITT in the acute phase is mainly based on administration of immunomodulatory agents.^15^ Immunomodulation by intravenous immunoglobulins (IVIGs) is effective but also expensive, and global shortages of IVIG have been well-documented even prior to the COVID-19 pandemic.^16,17^ In LMICs, access to these therapies might be limited, which could lead to a worse outcome for patients with CVST-VITT.

## Aims and/or hypothesis

The aims of this study were to gain insight into rates and characteristics of CVST-VITT reported from LMICs and to determine if its clinical manifestations, treatment, and outcome differ from CVST-VITT cases reported from HICs.

## Methods

Data were collected as part of an international observational study on CVST after COVID-19 vaccination. Details about the study methods have been published.^2,15^ Briefly, participating investigators were instructed to report consecutive cases of CVST developing within 4 weeks after any COVID-19 vaccine. For this particular study, we purposefully approached investigators from LMICs on multiple occasions through the International Cerebral Venous Thrombosis Consortium with the request to extend the invitation to as many colleagues in their country as possible. Detailed information on the search for eligible patients in the participating middle-income countries (MICs) is provided in Supplemental Table 2. Relevant patient data were extracted from the medical records.

The Medical Ethical Review Committee of Amsterdam University Medical Centers gave a waiver for formal approval of this study (reference W21_171#21.186). Informed consent was obtained from the patient or patient’s relatives if required by local law and/or hospital regulations. For this analysis, we included all data collected until 15 August 2022.

CVST cases were categorized into definite, probable, possible, or unlikely VITT based on the criteria from Pavord et al.^3^ (Supplemental Table 3). We considered the criterion for positive anti-PF4 antibodies to be met if the treating physician indicated that the patient tested positive for anti-PF4 antibodies, regardless of the antibody test type. Thrombocytopenia was defined as a platelet count < 150 × 10^3^/µL. In the main analysis, we focused on the cases classified as definite, probable, or possible VITT. The CVST cases with unlikely VITT were explored in a sensitivity analysis to investigate if CVST-VITT cases had potentially been misclassified as unlikely VITT, due to insufficient diagnostic resources in LMICs.

Countries were classified as low-income country, MIC, or HIC according to the World Bank definition.^18^ Ethnicity and sex were as investigator reported. The date of CVST diagnosis was divided into three timeframes (until March 2021, April 2021, and May 2021 and onwards) based on the timing of the first publication on VITT, which included treatment recommendations.^1,15^ If follow-up data after the initial hospital discharge were unavailable, the modified Rankin Scale (mRS) score at discharge was considered the final follow-up data. Functional independence was defined as having an mRS score of 0–2. Because the mortality rate due to CVST-VITT declined over time,^19^ we performed a sensitivity analysis to compare outcomes of patients diagnosed with CVST-VITT after May 2021 from MICs and HICs.

We used descriptive statistics to report study outcomes. We calculated 95% confidence intervals (95% CI) for proportions using the Wilson score method. We compared patient characteristics using the Mann–Whitney U test for non-normally distributed numerical variables, the chi-square test for binary and nominal categorical variables, and the chi-square test for trend for ordinal categorical variables. Fisher’s exact test for binary categorical variables and the Fisher–Freeman–Halton test for nominal categorical variables were used if any cells had an expected count less than five. *p* values less than 0.05 were considered significant. Analyses were conducted using IBM SPSS Statistics for Windows, version 28.0.1.0 (IBM Corp, Armonk, NY) and RStudio version 1.3.1093 (RStudio, PBC, Boston, MA) using the “Hmisc” package.

## Results

Between 31 March 2021 and 15 August 2022, 228 cases of CVST within 4 weeks of COVID-19 vaccination were reported from 25 countries (Supplemental Figure 1 and Supplemental Table 4). Of these, 63 cases (28%) came from seven MICs, and 165 cases (72%) from 18 HICs. There were no cases from low-income countries. Cases from MICs originated from Brazil, China, India, Iran, Mexico, Pakistan, and Turkey.

Of the 63 CVST cases from MICs, 32 (51%) fulfilled the criteria for definite, probable, or possible VITT, compared with 103 of the 165 (62%) cases from HICs (*p* = 0.110). The median age of CVST-VITT patients from MICs at diagnosis was 26 years (IQR 20–37 years), compared with 47 years (IQR 32–58 years) for CVST-VITT patients from HICs (*p* < 0.001, Table 1). In both groups, most CVST-VITT patients were women (25/32 [78%] and 77/103 [75%], respectively). Most CVST-VITT patients from MICs were from the Asian ethnic group (59%), while most CVST-VITT patients from HICs were from the white ethnic group (96%). In 24 of the 32 (75%) patients from MICs and in 101 of the 103 (98%) patients from HICs, CVST-VITT occurred after an adenovirus-based COVID-19 vaccine. Only 5 of the 32 (16%) CVST-VITT cases from MICs could be classified as definite VITT, compared with 70 of the 103 (68%) of CVST-VITT cases from HICs (*p* < 0.001). Anti-PF4 antibodies were frequently not tested in patients from MICs (missing in 21/32 [66%] cases vs. 9/103 [9%] cases from HICs). D-dimer levels were not tested or were unknown in 5 of the 32 (16%) CVST-VITT cases from MICs compared with 6 of the 103 (6%) cases from HICs.

**Table 1.** Characteristics of definite, probable, and possible CVST-VITT cases from middle- and high-income countries at presentation.

Characteristics	CVST-VITT cases from middle-income countries (N = 32)	CVST-VITT cases from high-income countries (N = 103)	*p* Value
Baseline characteristics, n/N (%)			
Age, median (IQR)	26 (20–37)	47 (32–58)	**<0.001**
Female sex	25/32 (78)	77/103 (75)	0.699
Ethnicity			
Asian	19/32 (59)	4/102 (4)	<0.001*
Black	0/32	0/102	
Hispanic	2/32 (6)	0/102	
White	4/32 (13)	98/102 (96)	
Other	7/32 (22)	0/102	
VITT classification			
Definite	5/32 (16)	70/103 (68)	**<0.001**
Probable	14/32 (44)	18/103 (17)	
Possible	13/32 (41)	15/103 (15)	
COVID-19 vaccine			
ChAdOx1 nCoV-19	24/32 (75)^a^	91/103 (88)	<0.001*
Ad26.COV2.S	0/32	10/103 (10)	
BBIBP-CorV	4/32 (13)	0/103	
Sinovac	4/32 (13)	0/103	
BNT162b2	0/32	2/103 (2)	
Conventional CVST risk factors			
Oral contraceptives	1/25 (4)	13/77 (17)	0.179*
Pregnancy/recent delivery^b^	1/25 (4)	0/77	0.245*
Infection	0/32	7/103 (7)	0.197*
Previous thromboembolism	0/32	2/103 (2)	>0.990*
Thrombophilia	1/32 (3)	1/103 (1)	0.419*
Cancer^c^	0/32	5/103 (5)	0.339*
Days from vaccination to symptom onset, median (IQR)	9 (4–12)	9 (7–11)^d^	0.870
Days from symptom onset to diagnosis, median (IQR)	3 (2–8)	3 (1–5)^e^	0.232
Time period of CVST diagnosis			
Until March 2021	0/32	40/103 (39)	**<0.001**
April 2021	1/32 (3)	25/103 (24)	
May 2021 and onwards	31/32 (97)	38/103 (37)	
Focal neurologic deficits	17/29 (59)	58/103 (56)	0.824
Coma	5/28 (18)	23/101 (23)	0.577
Seizure	5/30 (17)	15/103 (15)	0.775*
Concomitant VTE	3/31 (10)	26/97 (27)	**0.047**
Imaging modality used for diagnosis^f^			
CT-venography	5/32 (16)	85/103 (83)	**<0.001**
MRI	26/32 (81)	46/103 (45)	**<0.001**
MR-venography	27/32 (84)	43/103 (42)	**<0.001**
Digital subtraction angiography	1/32 (3)	8/103 (8)	0.686*
Intracranial hemorrhage	19/29 (66)	70/103 (68)	0.804
Laboratory data, n/N (%)			
Thrombocytopenia at any time during admission	31/32 (97)	99/103 (96)	>0.990*
Platelet count at presentation, median (IQR), ×103/µL	80 (41–128)	50 (28–77)	**0.020**
Platelet count nadir, median (IQR), ×103/µL	65 (36–115)	33 (18–55)	**0.001**
Anti-PF4 antibodies			
Positive	7/32 (22)	87/103 (84)	<0.001*
Negative	4/32 (13)	7/103 (7)	
Not tested or unknown	21/32 (66)	9/103 (9)	
D-dimer level (highest value)			
>4 µg/mL FEU	26/32 (81)	82/103 (80)	
2–4 µg/mL FEU	1/32 (3)	11/103 (11)	
<2 µg/mL FEU	0/32	4/103 (4)	
Not tested or unknown	5/32 (16)	6/103 (6)	

Significant *p* values are in bold. CVST: cerebral venous sinus thrombosis; FEU: fibrinogen equivalent units; IQR: interquartile range; PF4: platelet factor 4; VITT: vaccine-induced immune thrombotic thrombocytopenia; VTE: venous thromboembolism; CT: computed tomography; MRI: magnetic resonance imaging.

a Thirteen cases after Covishield (Serum Institute of India) vaccination and 11 cases after Vaxzevria (Oxford–AstraZeneca) vaccination.

b Within 12 weeks.

c In last 10 years.

d Two missing values.

e Two missing values.

f Multiple possible.

* Fisher’s exact test or Fisher–Freeman–Halton test.

CVST-VITT patients from MICs were diagnosed in a later time period compared with patients from HICs (1/32 [3%] cases vs. 65/103 [63%] cases with CVST diagnosis before May 2021, respectively, Table 1). The median interval between vaccination and symptom onset, and between symptom onset to diagnosis, did not differ between groups. Concomitant venous thromboembolism (VTE) at hospital presentation was less common (3/31 [10%] vs. 26/97 [27%], *p* = 0.047), and the median platelet count at presentation was higher (80 × 10^3^/µL [IQR 41–128] vs. 50 × 10^3^/µL [IQR 28–77], *p* = 0.020) in the MICs group than in the HICs group. The nadir platelet count was also higher in cases from MICs than that in cases from HICs (65 × 10^3^/µL [IQR 36–115] vs. 33 × 10^3^/µL [IQR 18–55], *p* = 0.001). In MICs, 14 of 27 (52%) patients were treated with a non-heparin as the first anticoagulant, compared with 56 of 90 (62%) patients in HICs (Table 2). The proportion of CVST-VITT patients who were treated with IVIG did not differ (19/30 [63%] vs. 63/99 [64%]).

**Table 2.** Treatment and outcomes of definite, probable, and possible CVST-VITT cases from middle- and high-income countries.

	CVST-VITT cases from middle-income countries (N = 32)	CVST-VITT cases from high-income countries (N = 103)	*p* value
**Treatment data, n/N (%)**			
Any anticoagulant treatment	27/31 (87)	90/103 (87)	>0.990*
Non-heparin as first anticoagulant^a^	14/27 (52)	56/90 (62)	0.335
Any immunomodulatory treatment^b^	19/30 (63)	68/99 (69)	0.584
Intravenous immunoglobulin	19/30 (63)	63/99 (64)	0.976
Plasma exchange	1/30 (3)	5/99 (5)	>0.990*
Corticosteroids	10/30 (33)	29/99 (29)	0.673
Eculizumab	0/30	2/99 (2)	>0.990*
Rituximab	0/30	1/99 (1)	>0.990*
Platelet transfusion	5/30 (17)	27/99 (27)	0.239
Endovascular treatment	2/32 (6)	15/102 (15)	0.360*
Decompressive neurosurgery	6/31 (19)	29/103 (28)	0.328
Intensive care unit admission	24/32 (75)	81/100 (81)	0.464
**Clinical events during admission, n/N (%)**			
New concomitant VTE	1/30 (3)	16/96 (17)	0.071*
Bleeding complication	8/29 (28)	36/101 (36)	0.419
Major bleeding^c^	6/28 (21)	30/100 (30)	0.373
**Discharge, n/N (%)**			
Duration hospital admission, median (IQR)	8 (4–12)^d^	7 (2–17)^e^	0.991
Discharge disposition			<0.001*
Home	22/31 (71)	34/101 (34)	
Rehabilitation center	1/31 (3)	21/101 (21)	
Other hospital	1/31 (3)	2/101 (2)	
Deceased	7/31 (23)	44/101 (44)	

Significant *p* values are in bold. CVST: cerebral venous sinus thrombosis; VITT: vaccine-induced immune thrombotic thrombocytopenia; VTE: venous thromboembolism.

a No low-molecular-weight heparin or unfractionated heparin.

b Multiple possible.

c According to the criteria of the International Society on Thrombosis and Haemostasis.

d Four missing values.

e Two missing values.

* Fisher’s exact test or Fisher–Freeman–Halton test.

A new concomitant VTE diagnosed during hospitalization was reported in 1 of 30 (3%) CVST-VITT patients from MICs and in 16 of 96 (17%) patients from HICs. Major bleeding events occurred in 6 of 28 (21%) patients from MICs during hospitalization compared with 30 of 100 (30%) patients from HICs. The in-hospital mortality rate was lower in the MICs group than in the HICs group (7/31 [23%, 95% CI 11–40] vs. 44/102 [43%, 95% CI 34–53], *p* = 0.039). In both the MICs group and the HICs group, one additional patient died after hospital discharge (Supplemental Figure 2). Mortality at 30 days after CVT diagnosis was 7 of 22 (32%) in MICs versus 43 of 100 (43%) in HICs. Most patients with a missing vital status at 30 days after diagnosis were discharged from the hospital before the 30-day mark without any follow-up (in 7/10 cases with a missing vital status in MICs and 2/3 cases in HICs). Functional independence at the latest follow-up was achieved by 21 of 29 (72%) CVST-VITT patients from MICs compared with 50 of 102 (49%) patients from HICs (*p* = 0.026).

When comparing patients with CVST-VITT who were diagnosed after May 2021, the in-hospital mortality rate in the MICs group was 7 of 30 (23%) compared with 12 of 38 (32%) in the HICs group (*p* = 0.452). In MICs, 21 of 30 (70%) CVST-VITT patients were discharged home compared with 16 of 38 (42%) patients in HICs (*p* = 0.022). Functional independence at the latest follow-up was achieved by 20 of 28 (71%) patients from MICs and 23 of 38 (61%) patients from HICs (*p* = 0.358).

CVST patients from MICs with unlikely VITT had a similar clinical profile to unlikely VITT patients from HICs (Supplemental Tables 5 and 6). Thrombocytopenia was present in 2 of 30 (7%) and 4 of 62 (6%) CVST patients with unlikely VITT from MICs and HICs, respectively. Patients with unlikely VITT from both MICs and HICs infrequently had a concomitant VTE at baseline (2/29 [7%] and 2/61 [3%]) and had low mortality rates at follow-up (2/31 [6%, 95% CI 2–21] vs. 4/62 [6%, 95% CI 3–15]).

## Discussion

The main results of this study are (1) CVST-VITT cases were less often reported from MICs than from HICs; (2) cases from MICs less often could be classified as definite VITT, mostly because anti-PF4 antibodies were not tested; (3) clinical presentation of CVST-VITT cases was comparable between MICs and HICs, except that patients from MICs were younger and less often had a concomitant VTE at presentation; (4) frequencies of use of IVIG and non-heparin anticoagulants were similar between MICs and HICs; and (5) mortality rate due to CVST-VITT was lower among cases from MICs than among cases from HICs.

The low reporting rate of CVST cases after COVID-19 vaccination from LMICs is remarkable since, by first of July 2022, 9.61 billion COVID-19 vaccines were administered in LMICs compared with 2.51 billion COVID-19 vaccines in HICs.^5^ In India alone, 1.67 billion ChAdOx1 nCoV-19 (COVISHIELD, Serum Institute of India) vaccines have been administered by 23 August 2022.^9^ Theoretically, CVST-VITT might be underdiagnosed or underreported in LMICs because of more limited access to imaging and laboratory tests and poorer quality of and access to health care. However, if the risk of CVST-VITT is the same across populations, that is, around one per 250,000 ChAdOx1 nCoV-19 vaccinations,^6,20^ thousands of cases should have occurred in India alone by now. It is unlikely that under-ascertainment and underreporting alone can fully explain the apparently strikingly lower incidence of CVST-VITT in LMICs. Even more so because CVST not related to COVID-19 vaccinations is more common in LMICs in the Middle East and South Asia than in HICs,^21^ and neurologists in these regions generally have ample experience with diagnosing CVST.^22^ Instead, these observations, in combination with the low reporting rate outside this study, could indicate that the risk of CVST-VITT differs between populations. This hypothesis is in line with findings from a study based on data from the AstraZeneca global safety database for the AZD1222 (ChAdOx1 nCoV-19) vaccine.^23^ The reporting rate of thrombosis with thrombocytopenia in this study was 88 times higher in Nordic countries than in Asian countries and Brazil, suggesting differences in the risk of thrombosis with thrombocytopenia after AZD1222 vaccination between the observed populations. There could be several explanations for a difference in risk of CVST-VITT between populations. First, there could be a genetic predisposition for VITT that varies across ethnicities, as has been described for the association between narcolepsy and the Pandemrix H1 N1 vaccine.^14,24^ Second, environmental factors could play a role, for instance, exposure to certain viruses or medications in the general population. Third, it has been proposed that manufacturing differences between batches of vaccines or different factories may explain discrepancies in rates of VITT.^25^ The consistency of the low rates of VITT reported from LMICs over time across multiple regions, however, makes this less likely, and no evidence to date has supported this hypothesis. Unfortunately, as far as the authors are aware of, no (inter)national pharmacovigilance data are available besides the data from the AstraZeneca global safety database to confirm the low incidence of CVST-VITT in LMICs.

The results of this study can be of value to health care policy-makers to evaluate the role of adenovirus-based COVID-19 vaccines in vaccination campaigns in LMICs, which are still in full swing. As of June 2022, only 58 of 194 (30%) World Health Organization member states had reached the target of 70% vaccination coverage.^10^ Moreover, further investigations into the possible difference in the incidence of CVST-VITT across populations may help to shed further light on the pathophysiology of VITT and possibly similar conditions such as heparin-induced thrombocytopenia.

Anti-PF4 antibodies and, to a lesser extent, D-dimer levels were often not tested in CVST cases from MICs. Without these tests, a suspected CVST-VITT patient cannot meet the criteria for definite or probable VITT.^3^ These findings suggest that the VITT criteria by Pavord et al.^3^ might be less suitable for MICs. A more clinically based case definition for vaccine-related CVST such as the combination of CVST with new-onset thrombocytopenia within 28 days after COVID-19 vaccination, as proposed by the Brighton Collaboration,^26^ might be more suitable for MICs. Nevertheless, the clinical profile of unlikely VITT patients was very different from that of definite, probable, and possible CVST-VITT patients in both MICs and HICs, indicating that unlikely VITT patients in MICs were indeed non-VITT CVST patients instead of underdiagnosed CVST-VITT patients. In addition, the functional outcomes of the unlikely CVST-VITT patients from MICs and HICs were comparable to functional outcomes of CVST patients not related to vaccination, suggesting that these were spurious cases not related to vaccination.^27^

Although clinical characteristics of CVST-VITT cases were largely comparable between MICs and HICs, some differences between both groups should be noted. First, patients from MICs were younger than those from HICs, which is probably explained by differences in age groups that received adenoviral COVID-19 vaccines due to age restrictions on the use of these vaccines in many HICs.^6^ Second, concomitant VTE at presentation was less frequently reported in cases from MICs than in those from HICs. Possible explanations for this difference might be under-ascertainment of subclinical thrombosis in MICs and less extensive screening for thrombosis at other sites, younger median age, or a true lower rate of VTE in cases from MICs.^28^ Third, the platelet count at presentation and the platelet count nadir were higher in MICs than in HICs. Theoretically, this might indicate that the general severity of CVST-VITT is lower in cases from MICs. This might be explained by the fact that CVST-VITT patients from MICs were diagnosed in a later time period than patients from HICs, when there was already global awareness and guidelines regarding diagnosis and treatment of CVST-VITT.^15^ However, the time from symptom onset to diagnosis and frequency of IVIG administration, non-heparin anticoagulants use, and platelet transfusions were similar between MICs and HICs.

The observation that treatment of CVST-VITT cases did not differ between MICs and HICs is important because it suggests that knowledge on how to treat this condition is well disseminated in MICs and that access to, for example, IVIG is sufficient at the centers that contributed. Still, we should be careful to draw definitive conclusions on this issue, as the registry does not contain data on consecutive cases, or cases from low-income countries.

In-hospital mortality due to CVST-VITT was lower among patients from MICs than among those from HICs. This difference might again be related to the fact that CVST-VITT cases from MICs were younger. The difference could also be partially attributed to the fact that cases from MICs were diagnosed in a later time period, as was suggested by the sensitivity analysis including only the cases diagnosed after May 2021. Alternatively, very severe CVST-VITT cases—which sometimes succumb within hours of hospital admission—may have been underdiagnosed in MICs. In addition, the diagnostic workup of patients with an unexplained death in the weeks following COVID-19 vaccination might be more limited in some MICs, which could lead to under-ascertainment of CVST-VITT.

Our study has several limitations. Despite our multiple efforts to reach investigators from LMICs, local restrictions may have influenced the decision of investigators to participate in the study, and reporting within participating countries is almost certainly incomplete. We presume that our coverage of the poorest subpopulations of LMICs was likely worse than that of the more affluent parts of the population. Second, we did not receive any cases from low-income countries, so we cannot draw conclusions on the frequency and clinical manifestations of CVST-VITT in these countries. Third, all data collected in the study were gathered as part of routine medical care, and the data were not centrally adjudicated.

In conclusion, the absolute number of CVST-VITT cases reported from LMICs was low despite the widespread use of adenoviral COVID-19 vaccines in these countries, which is consistent with previous pharmacovigilance reporting regarding VITT^23^ and may possibly indicate that susceptibility of this condition varies between populations. Clinical presentation and treatment of CVST-VITT cases were largely similar in MICs and HICs, while mortality was lower in patients from MICs.

## Supplemental Material

sj-docx-1-wso-10.1177_17474930231182901 – Supplemental material for Cerebral venous sinus thrombosis due to vaccine-induced immune thrombotic thrombocytopenia in middle-income countries Click here for additional data file. Supplemental material, sj-docx-1-wso-10.1177_17474930231182901 for Cerebral venous sinus thrombosis due to vaccine-induced immune thrombotic thrombocytopenia in middle-income countries by Anita van de Munckhof, Afshin Borhani-Haghighi, Sanjith Aaron, Katarzyna Krzywicka, Mayte Sánchez van Kammen, Charlotte Cordonnier, Timothy J Kleinig, Thalia S Field, Sven Poli, Robin Lemmens, Adrian Scutelnic, Erik Lindgren, Jiangang Duan, Yıldız Arslan, Eric CM van Gorp, Johanna A Kremer Hovinga, Albrecht Günther, Katarina Jood, Turgut Tatlisumak, Jukka Putaala, Mirjam R Heldner, Marcel Arnold, Diana Aguiar de Sousa, Mohammad Wasay, Antonio Arauz, Adriana Bastos Conforto, José M Ferro and Jonathan M Coutinho in International Journal of Stroke
