# Supplementary material for: Cerebral venous sinus thrombosis due to vaccine-induced immune thrombotic thrombocytopenia in middle-income countries
Source: Int J Stroke. 2023 Jun 30;18(9):1112–20. doi: 10.1177/17474930231182901 (PMC10614174; doi:10.1177/17474930231182901)
Supplement: sj-docx-1-wso-10.1177_17474930231182901 – Supplemental material for Cerebral venous sinus thrombosis due to vaccine-induced immune thrombotic thrombocytopenia in middle-income countries [file sj-docx-1-wso-10.1177_17474930231182901.docx]

**SUPPLEMENTAL MATERIAL**

**Cerebral Venous Sinus Thrombosis due to Vaccine-Induced Immune Thrombotic Thrombocytopenia in Middle-Income Countries**

**Table of contents**

The Cerebral Venous Sinus Thrombosis with Thrombocytopenia Syndrome Study Group......................................2

Supplemental Table 1. Literature search....................................................................................................................4

Supplemental Table 2. Overview of case identification in participating middle-income countries............................5

Supplemental Table 3. Criteria for classification of vaccine-induced immune thrombotic thrombocytopenia..........7

Supplemental Table 4. Overview of cases per country………....................................................………...............…8

Supplemental Table 5. Characteristics of CVST cases with unlikely VITT from middle- and high-income countries.................................................................................................................................................................... 9

Supplemental Table 6. Treatment and outcomes of CVST cases with unlikely VITT from middle- and high-income countries................................................................................................................................................................. 11

Supplemental Figure 1. Flowchart of patient selection……………………………………………………………12

Supplemental Figure 2. mRS score at latest follow-up of CVST-VITT patients from middle-income countries and high-income countries .............................................................................................................................................13

Supplemental References ........................................................................................................................................14

**The Cerebral Venous Sinus Thrombosis with Thrombocytopenia Syndrome Study Group**

Joshua Mbroh,^1,2^ Alfonso Ciccone,^3^ Matthias Wittstock,^4^ Julian Zimmermann,^5^ Felix J Bode,^5^ Mona Skjelland,^6^ Vanessa Dizonno,^7^ Annemie Devroye,^8^ Sini Hiltunen,^9^ Marco Petruzzellis,^10^ Tamam Bakchoul,^11^ Marcel Levi,^12^ Saskia Middeldorp,^13^ Aarti R Sharma,^14^ Abdoreza Ghoreishi,^15^ Ahmed Elkady,^16^ Alberto Negro,^17^ Alexander Gutschalk,^18^ Alina Buture,^19^ Alvaro Cervera,^20^ Ana Paiva Nunes,^21^ Ana Romina Montané Baños,^22^ Andreas Tiede,^23^ Anil M Tuladhar,^24^ Annerose Mengel,^25^ Antonio Medina,^26^ Avinash Aujayeb,^27^ Balakrishnan Ramasamy,^28^ Barbara Casolla,^29^ Neil Spratt,^30^ Bentolhoda Ziaadini,^31^ Boby Varkey Maramattom,^32^ Brian Buck,^33^ Carlos Garcia-Esperon,^34^ Caroline Vayne,^35^ Christian Jacobi,^36^ Christian Pfrepper,^37^ Deepti Bal,^38^ Domenico Sergio Zimatore,^39^ Dominik Michalski,^40^ Dylan Blacquiere,^41^ Elias Johansson,^42,43^ Elisa Cuadrado-Godia,^44^ Elyar Sadeghi-Hokmabadi,^45^ Emmanuel Carrera,^46^ Emmanuel De Maistre,^47^ Espen Saxhaug Kristoffersen,^48^ Etrat Hooshmandi,^49^ Fabrice Bonneville,^50^ Fabrice Vuillier,^51^ Fabrizio Giammello,^52^ Florindo D’Onofrio,^53^ Georgios Tsivgoulis,^54^ Giosue Gulli,^55^ Hans Katzberg,^56^ Igor Sibon,^57^ Irem Baharoglu,^58^ Jaime Masjuan,^59^ João Fernandes,^60^ Johann Pelz,^40^ Jorge Octavio López Esparza,^61^ Judith Schouten,^62^ Karl Ng,^63^ Laurent Derex,^64^ Laurent Puy,^65^ Leila Poorsaadat,^66^ Lenise Valler,^67^ Letícia Januzi de Almeida Rocha,^68^ Luis Murillo-Bonilla,^69^ Lukas Kellermair,^70^ Mar Morin Martin,^71^ Maria Sofia Cotelli,^72^ Maria Hernandez Perez,^73^ Marialuisa Zedde,^74^ Mariana Carvalho Dias,^75^ Marta Carvalho,^76^ Masoud Ghiasian,^77^ Meenakshisundaram Umaiorubahan,^78^ Mehrdad Roozbeh,^79^ Michele Romoli,^80^ Miguel Miranda,^81^ Miriam Wronski,^63^ Mohammad Saadatnia,^82^ Monica Bandettini di Poggio,^83^ Mostafa Almasi-Dooghaee,^84^ Nahid Hoseininejad Mir,^85^ Nasli R Ichaporia,^86^ Naveen Kumar Paramasivan,^87^ Nicolas Raposo,^88^ Nima Fadakar,^49^ Nyika Kruyt,^89^ Olivier Detante,^90^ Pankaj Sharma,^91^ Paolo Candelaresi,^92^ Pasquale Scoppettuolo,^93^ Peggy Reiner,^94^ PN Sylaja,^87^ Ravi Kumar Karunakaran,^78^ Ricardo Vieira,^95^ Rolf Kern,^96^ Rudy Goh,^97^ Sapna Erat Sreedharan,^87^ Seán Murphy,^19^ Serge Timsit,^98^ Shelagh Coutts,^99^ Shyam S Sharma,^100^ Silvia Schoenenberger,^18^ Simon Nagel,^18,101^ Subhash Kaul,^102^ Theodoros Karapanayiotides,^103^ Thomas Gattringer,^104^ Thomas Mathew,^105^ Thorsten Bartsch,^106^ Vincenzo Palma,^107^ Zahra Mirzaasgari,^108^ Zohreh Zamani,^109^ Alireza Mirahmadizadeh,^110^ Alonso Gutierrez-Romero,^111^ Ivan Iván Valdes-Ferrer,^112^ Santa Elizabeth Ceballos-Liceaga,^113^ Ana Maria Santibañez-Copado^114^.

^1^Department of Neurology & Stroke, University Hospital Tuebingen, Eberhard-Karls University, Tuebingen, Germany, ^2^Hertie Institute for Clinical Brain Research, Eberhard-Karls University, Tuebingen, Germany, ^3^Department of Neurology, Carlo Poma Hospital, Azienda Socio Sanitaria Territoriale di Mantova, Mantua, Italy, ^4^University Hospital Rostock, Rostock, Germany, ^5^Universitätsklinikum Bonn, Bonn, Germany, ^6^Oslo University Hospital, Oslo, Norway, ^7^University of British Columbia, Vancouver, Canada, ^8^Department of Neurology, University Hospitals Leuven, Leuven, Belgium, ^9^Department of Neurology, Helsinki University Hospital and University of Helsinki, Helsinki, Finland, ^10^AOU Consorziale Policlinico di Bari, Bari, Italy, ^11^Institute for Clinical and Experimental Transfusion Medicine, Medical Faculty of Tuebingen, University Hospital of Tuebingen, Tuebingen, Germany, ^12^Department of Vascular Medicine, Amsterdam UMC, University of Amsterdam, Amsterdam, The Netherlands, ^13^Department of Internal Medicine & Radboud Institute of Health Sciences, Radboud University Medical Center, Nijmegen, The Netherlands, ^14^Imperial College School of Medicine, London, United Kingdom, ^15^Stroke Research Group, Head of Stroke Care Unit, Department of Neurology, Vali-e-Asr Hospital, School of Medicine, Zanjan University of Medical Sciences, Iran, ^16^Saudi German Hospital, Jeddah, Saudi Arabia, ^17^Ospedale del Mare, Naples, Italy, ^18^Department of Neurology, Heidelberg University Hospital, Heidelberg, Germany, ^19^Mater Misericordiae University Hospital, Dublin, Ireland, ^20^Royal Darwin Hospital, Tiwi, Australia, ^21^Stroke Centre, Lisbon Central University Hospital Centre, Lisbon, Portugal, ^22^Queretaro General Hospital, Santiago de Querétaro, Mexico, ^23^Hannover Medical School, Hannover, Germany, ^24^Radboud University Medical Center, Department of Neurology, Donders Center for Medical Neurosciences, Nijmegen, The Netherlands, ^25^University Hospital Tuebingen, Tuebingen, Germany, ^26^Hospital Universitario Nuestra Señora de Candelaria, Santa Cruz de Tenerife, Spain, ^27^Northumbria Healthcare NHS Foundation Trust, Cramlington, United Kingdom, ^28^PSG Institute of Medical Sciences and Research, Coimbatore, Tamil Nadu, India, ^29^Stroke Unit, Hôpital Pasteur 2, URRIS-UR2CA, Unité de Recherche Clinique Cote d'Azur, Cote d'Azur University, Nice, France, ^30^Department of Neurology, John Hunter Hospital, Newcastle, Australia, ^31^Neurology Research Center, Kerman University of Medical Sciences, Kerman, Iran, ^32^Aster Medcity, Kochi, Kerala, India, ^33^University of Alberta Hospital, Edmonton, Canada, ^34^John Hunter Hospital, New Lambton Heights, Australia, ^35^Tours University Hospital, Tours, France, ^36^Department of Neurology, Krankenhaus Nordwest, Frankfurt am Main, Germany, ^37^Leipzig University Hospital, Leipzig, Germany, ^38^Department of Neurosciences, Christian Medical College Hospital, Vellore, Tamil Nadu, India, ^39^Policlinico di Bari, Bari, Italy, ^40^Department of Neurology, University of Leipzig, Leipzig, Germany, ^41^The Ottawa Hospital, Ottawa, Canada, ^42^Department of Neurology, Sahlgrenska University Hospital, Gothenburg, Sweden and Department of Clinical Neuroscience, Institute of Neuroscience and Physiology, Sahlgrenska Academy at University of Gothenburg, Sweden, ^43^Department of Clinical Science, Umeå University, Umeå, Sweden, ^44^Hospital del Mar Medical Research Institute (IMIM), Barcelona, Spain, ^45^NeuroSciences Research Center (NSRC), Imam-Reza hospital, Tabriz University of Medical Sciences, Tabriz, Iran, ^46^Hôpitaux Universitaires de Genève, Geneva, Switzerland, ^47^CHU Dijon, Dijon, France, ^48^Department of Neurology, Akershus University Hospital, Lorenskog, Norway, ^49^Clinical Neurology Research Center, Shiraz University of Medical Sciences, Shiraz, Iran, ^50^Toulouse University Hospital, Toulouse, France, ^51^University Hospital of Besancon, Besancon, France, ^52^Translational Molecular Medicine and Surgery 36th Cycle, Department of BIOMORF, Stroke Unit, Department of Clinical and Experimental Medicine, University Hospital G. Martino, Messina, Italy, ^53^University Hospital G. Martino, Messina, Italy, ^54^Second Department of Neurology, National & Kapodistrian University of Athens, School of Medicine, Athens, Greece, ^55^Ashford and St Peters Hospital NHS Foundation Trust, Surrey, United Kingdom, ^56^Toronto General Hospital, Toronto, Canada, ^57^Bordeaux University Hospital, Bordeaux, France, ^58^Haga Hospital, The Hague, The Netherlands, ^59^Ramón y Cajal Hospital, Madrid, Spain, ^60^Norra Älvsborgs Länssjukhus, Trollhättan, Sweden, ^61^Centenario Hospital Miguel Hidalgo, Aguascalientes, México, ^62^Rijnstate Hospital Arnhem, Arnhem, The Netherlands, ^63^Department of Neurology, Royal North Shore Hospital, Sydney, Australia, ^64^Hospices Civils de Lyon, Lyon, France, ^65^Univ. Lille, Inserm, CHU Lille, U1172 - LilNCog - Lille Neuroscience & Cognition, F-59000 Lille, France, ^66^Department of Neurology, Arak University of Medical Sciences, Arak, Iran, ^67^UNICAMP Universidade Estadual de Campinas, Campinas, Brazil, ^68^Universidade Federal de Alagoas (Federal University of Alagoas - in English), Maceió, Brazil, ^69^Instituto Panvascular de Occidente, Guadalajara, Jalisco, México, ^70^Johannes Kepler University Linz, Linz, Austria, ^71^Hospital complex of Toledo, Toledo, Spain, ^72^Neurology Unit ASST Valcamonica, Esine, Brescia-Italy, ^73^Stroke Unit, Department of Neurosciences, Germans Trias i Pujol University Hospital, Badalona, Spain, ^74^Azienda Unità Sanitaria Locale-IRCCS di Reggio Emilia, Reggio Emilia, Italy, ^75^Department of Neurosciences and Mental Health, Hospital de Santa Maria, Centro Hospitalar Universitario Lisboa Norte, University of Lisbon, Lisbon, Portugal, ^76^Department of Neurology, Centro Hospitalar Universitario São João and Department of Clinical Neurosciences and Mental Health, Faculty of Medicine, University of Porto, Portugal, ^77^Sina Hospital, Hamadan University of Medical Sciences, Hamadan, Iran, ^78^Institute of Neurosciences, SIMS hospital, Chennai, Tamil Nadu, India, ^79^Brain Mapping Research Center, Shahid Beheshti University of Medical Sciences, Tehran, Iran, ^80^Neurology and Stroke Unit, Department of Neuroscience, Bufalini Hospital, Cesena, Italy, ^81^Hospital de Cascais Dr. José de Almeida, Cascais, Portugal, ^82^Isfahan Neurosciences Research Center, Isfahan University of Medical Sciences, Isfahan, Iran, ^83^IRCSS Ospedale Policlinico San Martino, Genoa, Italy, ^84^Firoozgar hospital, School of Medicine, Iran University of Medical sciences, Tehran, Iran, ^85^Department of Neurology, Lorestan University of Medical Sciences, Khorramabad, Iran, ^86^Sahyadri Superpeciality Hospital, Pune, Maharashtra, India, ^87^Sree Chitra Tirunal Institute for Medical Sciences and Technology, Trivandrum, Kerala, India, ^88^Department of neurology, Centre Hospitalier Universitaire de Toulouse, Toulouse, France, ^89^Leiden University Medical Centre, Leiden, The Netherlands, ^90^Department of Neurology, CHU Grenoble Alpes, Grenoble, France, ^91^Institute of Cardiovascular Disease, Royal Holloway University of London, London, United Kingdom, ^92^Neurology and Stroke Unit, Cardarelli Hospital, Naples, Italy, ^93^Department of Neurology, Cliniques Universitaires Saint-Luc, Université Catholique de Louvain, Hippocrate 10, 1200, Brussels, Belgium, ^94^Lariboisière Hospital, Neurology Department, Assistance Publique Hopitaux de Paris, France, ^95^Universidade Federal do Cariri, Juazeiro do Norte, Brazil, ^96^Kempten Hospital, Kempten, Germany, ^97^Royal Adelaide Hospital, Adelaide, Australia, ^98^Department of Neurology & stroke unit, Hôpital de la Cavale Blanche, CHRU de Brest (University Hospital), Université de Bretagne Occidentale, Inserm1078, Brest, France, ^99^Foothills Medical Centre, Calgary, Canada, ^100^Edinburgh Medical School, University of Edinburgh, Edinburgh, Scotland, ^101^Department of Neurology, Klinikum der Stadt Ludwigshafen gGmbH, Ludwigshafen, Germany, ^102^KIMS Hospital, Hyderabad, Telangana, India, ^103^2nd Department of Neurology, Aristotle University of Thessaloniki, School of Medicine, AHEPA University Hospital, ^104^Department of Neurology and Division of Neuroradiology, Vascular and Interventional Radiology, Department of Radiology, Medical University of Graz, Austria, ^105^St John's Medical College Hospital, Bengaluru, Karnataka, India, ^106^University Medical Center Schleswig-Holstein, Campus Kiel, Germany, ^107^Department of Neurology, Stroke Unit, Ospedale del Mare, ASL Napoli 1 Centro, Napoli, Italy, ^108^Department of Neurology, Firoozgar Hospital, School of Medicine, Iran University of Medical Sciences, Tehran, Iran, ^109^Department of Neurology, Firoozabadi Hospital, Iran University of Medical sciences, Tehran, Iran, ^110^Department of Epidemiology, Shiraz University of Medical Sciences, Shiraz, Iran, ^111^Stroke Clinic, Instituto Nacional de Neurología y Neurocirugía Manuel Velasco Suarez, Mexico City, Mexico, ^112^Neurology Department, Instituto Nacional de Ciencias Medicas y Nutrición Salvador Zubiran, Mexico City, Mexico, ^113^Dirección General de Epidemiología, Secretaria de Salud, Guerrero, Mexico, ^114^Centro Nacional para la Salud de la Infancia y Adolescencia, Secretaria de Salud, Mexico City, Mexico

**Supplemental Table 1. Literature search.**

| **Aim search** | We searched Pubmed on December 15, 2022 for studies reporting CVST after a recent COVID-19 vaccination in LMICs. |
| --- | --- |
| **Search terms** | The following search terms were used: ((sinus*[TI] AND thrombosis[TI]) OR (thrombosis[TI] AND cerebral[TI] AND (venous[TI] OR vein*[TI] OR sinus*[TI])) OR ("Sinus Thrombosis, Intracranial"[MESH]) OR (intracranial[TI] AND thrombosis[TI])) AND ("COVID-19 Vaccines"[Mesh] OR (vaccin*[TIAB] AND (COVID-19[TIAB] OR "SARS-CoV-2"[TIAB] OR corona*[TIAB])) OR VITT[TIAB] OR TTS[TIAB]). |
| **Findings** | This search resulted in 169 articles. The titles, abstracts, and author affiliations were screened. Studies reporting original data on CVST cases after COVID-19 vaccination from LMICs were eligible for inclusion. We excluded reports from HICs, studies that did not report original data, and studies about CVST not related to vaccination. Sixteen studies met the eligibility criteria. All studies were from MICs, no studies from low-income countries were identified. In these 16 studies, 18 individual cases with confirmed CVST were described. Of these, four cases had definite VITT according to the Pavord criteria,^1^ four cases had probable VITT, and one had possible VITT. Eight cases (44%) had unlikely VITT. For one case, no details were available to classify VITT. All nine cases with definite, probable, or possible VITT occurred after ChAdOx1 nCoV-19 vaccination. No studies comparing patient-level data between different populations have been published so far. |

CVST = cerebral venous sinus thrombosis; HICs = high-income countries; LMICs = low- and middle-income countries; MICs = middle-income countries; VITT = vaccine-induced immune thrombotic thrombocytopenia.

**Supplemental Table 2. Overview of case identification in participating middle-income countries.**

| **Country** | **National Coordinator** | **Recruitment^a^** | **Catchment area^a^** | **Number of vaccines administered per country until August 2022** |
| --- | --- | --- | --- | --- |
| Brazil | Prof. Adriana Conforto | The National Coordinator reached out to Brazilian investigators by sending emails to all members of the Brazilian Stroke Organization. In the center of the National Coordinator, cases were consecutively collected. In other centers, cases were identified based on recall. | Catchment area participating centers:   1. Hospital das Clínicas da Faculdade de Medicina da Universidade de São Paulo:  22 million 2. University of Campinas:   6.5 million   1. Hospital Universitário Professor Alberto Antunes:   3 million  Population Brazil: ~214 million.^2^ | - Butantan/Sinovac:   110 million vaccines   - Fiocruz/AstraZeneca:   149 million vaccines   - Janssen:   29 million vaccines   - Pfizer/Biontech:   200 million vaccines^3^ |
| China | Prof. Jiangang Duan | Cases were centrally collected in the ward of the National Coordinator in China (Xuanwu hospital affiliated to Capital Medical University). Patients with CVST were consecutively collected and included in the study when they met the inclusion criteria. | Catchment area unknown.  Xuanwu hospital has around 1159 beds and provides outpatient services to 8000 patients every day.^4^ The Department of Neurology of Xuanwu Hospital has 200 beds and treats 600,000 outpatients annually.^5^ | Not available |
| India | Prof. Sanjith Aaron | The National Coordinator reached out to 247 neurologists across India through WhatsApp groups. This covered all the secondary and tertiary referral centers in India. All cases were collected by the National Coordinator. In addition, samples of suspected cases were sent to the center of the National Coordinator to test for PF4 antibodies.  Within the participating centers, cases were identified the following way:   - Aster Medcity:   Consecutive cases and based on recall   - CMC Vellore:   CVST registry   - Sahyadri Hospital:   Based on recall   - PSG Hospitals Coimbatore:   Medical records   - KIMS Hospitals Secunderabad:   Medical records   - SIMS Hospital Chennai:   Based on recall   - SCTIMST:   Stroke registry   - St. John's Medical College Hospital:   Medical records | All the hospitals are quaternary care referral centers – the approximate catchment area of each center is as follows:   - Aster Medcity and SCTIMST together:   35 million   - CMC Vellore:   140 million   - Sahyadri Hospital:   6.2 million   - PSG Hospitals Coimbatore and SIMS Hospital Chennai:   72 million   - KIMS Hospitals Secunderabad:   12 million   - St. John's Medical College Hospital:   60 million  Population India: ~1.41 billion.^2^ | - ChAdOx1 nCoV-19 (COVISHIELD):   1.67 billion^6^ |
| Iran | Prof. Afshin Borhani-Haghighi | A survey was sent by the National Coordinator to all 1400 members of the Iranian Neurological Association. Neurologists were asked to search their hospital records for eligible patients using primary diagnostic ICD-10 codes G08 (intracranial and intraspinal phlebitis and thrombophlebitis), O87.3 (cerebral venous thrombosis in the puerperium), I63.6 (cerebral infarction due to cerebral venous thrombosis, nonpyogenic), I67.6 (nonpyogenic thrombosis of the intracranial venous system). Case report forms were centrally collected by the National Coordinator. | Estimated catchment area based on the population of different provinces according to the 2016 census.   \| **Province** \| **Population** \| \| --- \| --- \| \| Tehran \| 13.3 million \| \| Fars \| 4.9 million \| \| Esfahan \| 5.1 million \| \| Lorestan \| 1.8 million \| \| Ghazvin \| 1.3 million \| \| Zanjan \| 1 million \| \| Markazi \| 1.4 million \| \| Kerman \| 3.2 million \| \| Bushehr \| 1.2 million \| \| Kerman-shahan \| 2.0 million \|   Population Iran: ~88 million.^2^ | - COVID-19 vaccine (all types):   153.67 million^7^  Exact numbers per type of vaccine are not available. Based on data from one province,^8^ the estimated number of vector-based vaccines is around 13 million. |
| Mexico | Prof. Antonio Arauz | The Mexican Ministry of Health collects information on all adverse events following immunization (AEFI) through a passive surveillance system, including no less than 23,300 public and private medical units distributed across the country.  After detection of potentially serious neurologic AEFIs following SARS-CoV-2 vaccinations, the Mexican Ministry of Health appointed an ad-hoc committee consisting of five experienced neurologists and a neuroradiologist (among which the National Coordinator of the study) to perform a thorough analysis of every potentially serious neurologic AEFI. During this period, more than 90 million doses of six different SARS-CoV-2 vaccines were administered in Mexico, for which the Mexican Epidemiological Surveillance System received and processed more than 29,000 AEFI reports. Among those, (98%) were classified as non-serious, and (2%) as serious. | Catchment area unknown. The hospital of the National Coordinator (Instituto Nacional de Neurología y Neurocirugía) offers close to 90,000 medical consultations per year.^9^  Population Mexico: ~127 million.^2^ | Not available |
| Pakistan | Prof. Mohammed Wasay | The National Coordinator started a national CVST registry. Cases were centrally and consecutively collected at the center of the National Coordinator (Aga Khan University). In addition, eligible cases were identified through hospital record systems. | The catchment area of Aga Khan University is 30-35 million people.  Population Pakistan: ~231 million.^2^ | - COVID-19 vaccine (all types):   278 million^10^ |
| Turkey | Dr. Yıldız Arslan | Not available | Not available | Not available |

AEFI = adverse event following immunization; CVST = cerebral venous sinus thrombosis; ICD-10 = International Classification of Diseases 10th Revision; PF4 = platelet factor 4.

^a^Data are as provided by the local investigators unless indicated otherwise.

**Supplemental Table 3. Criteria for classification of vaccine-induced immune thrombotic thrombocytopenia.**

| Criteria | |
| --- | --- |
| 1. Presence of thrombosis; | |
| 1. Onset of symptoms 5–30 days after COVID-19 vaccination; | |
| 1. Thrombocytopenia defined as a platelet count <150 x10^3^/µL^a^; | |
| 1. D-dimer level >4 µg/mL FEU^b^; | |
| 1. Positive anti-PF4 antibody test. | |
| Classification of VITT | |
| Definite VITT | All VITT criteria fulfilled |
| Probable VITT | D-dimer level >4 µg/mL FEU, and three other criteria fulfilled  OR  D-dimer level 2-4 µg/mL FEU or unknown, and all other four criteria fulfilled |
| Possible VITT | D-dimer level >4 µg/mL FEU, and two other criteria fulfilled  OR  D-dimer level 2-4 µg/mL FEU or unknown, and three other criteria fulfilled  OR  D-dimer level <2 µg/mL FEU, but all other four criteria fulfilled |
| Unlikely VITT | D-dimer level >4 µg/mL FEU, and one or zero other criteria fulfilled  OR  D-dimer level 2-4 µg/mL FEU or unknown, and two or less other criteria fulfilled  OR  D-dimer level <2 µg/mL FEU, and three or less other criteria fulfilled |

FEU = fibrinogen-equivalent unit; PF4 = platelet factor 4; VITT = vaccine-induced immune thrombotic thrombocytopenia.

Based on the criteria from an expert hematology panel by the British Society for Haematology, as described by Pavord et al. (2021).^1, 11^

^a^To convert to 10^9^/L, multiply values by 1; ^b^To convert to nmol/L, multiply values by 5·476.

**Supplemental Table 4. Overview of cases per country.**

|  | Country | Number of cases |
| --- | --- | --- |
| **Middle-income countries** | Brazil | 5 |
|  | China | 4 |
|  | India | 19 |
|  | Iran | 25 |
|  | Mexico | 5 |
|  | Pakistan | 4 |
|  | Turkey | 1 |
|  | **Total** | **63** |
| **High-income countries** | Australia | 11 |
|  | Austria | 3 |
|  | Belgium | 6 |
|  | Canada | 10 |
|  | Finland | 4 |
|  | France | 27 |
|  | Germany | 35 |
|  | Greece | 2 |
|  | Ireland | 3 |
|  | Italy | 17 |
|  | The Netherlands | 7 |
|  | Norway | 7 |
|  | Portugal | 9 |
|  | Saudi Arabia | 3 |
|  | Spain | 11 |
|  | Sweden | 3 |
|  | Switzerland | 2 |
|  | United Kingdom | 5 |
|  | **Total** | **165** |

**Supplemental Table 5. Characteristics of CVST cases with unlikely VITT from middle- and high-income countries.**

|  | **Unlikely CVST-VITT cases from middle-income countries**  **(N = 31)** | **Unlikely CVST-VITT cases from high-income countries**  **(N = 62)** |
| --- | --- | --- |
| **Baseline characteristics, n/N (%)** |  |  |
| Age at diagnosis, median (IQR), years | 39 (32-51) | 50 (35-66) |
| Female sex | 11/31 (35) | 49/62 (79) |
| Ethnicity |  |  |
| Asian | 11/31 (35) | 3/62 (5) |
| Black | 0/31 | 3/62 (5) |
| Hispanic | 3/31 (10) | 0/62 |
| White | 11/31 (35) | 55/62 (89) |
| Other | 6/31 (19) | 1/62 (2) |
| COVID-19 vaccine |  |  |
| ChAdOx1 nCoV-19 | 11/31 (35)^a^ | 22/62 (35)^b^ |
| Ad26.COV2.S | 0/31 | 0/62 |
| BBIBP-CorV | 6/31 (19) | 0/62 |
| Sinovac | 9/31 (29) | 0/62 |
| BNT162b2 | 3/31 (10) | 35/62 (56) |
| mRNA-1273 | 0/31 | 5/62 (8) |
| Other | 2/31 (6) | 0/62 |
| Conventional CVST risk factors |  |  |
| Oral contraceptives | 5/11 (45) | 17/49 (35) |
| Pregnancy / recent delivery^c^ | 0/11 | 1/49 (2) |
| Infection | 0/31 | 1/62 (2) |
| Previous thromboembolism | 0/31 | 2/62 (3) |
| Thrombophilia | 3/31 (10) | 2/62 (3) |
| Cancer^d^ | 0/31 | 3/62 (5) |
| Days from vaccination to symptom onset, median (IQR) | 11 (4-18) | 8 (3-16) |
| Days from symptom onset to diagnosis, median (IQR) | 3 (2-7) | 3 (1-7) |
| Time period of CVST diagnosis |  |  |
| Until March 2021 | 2/31 (6) | 16/62 (26) |
| April 2021 | 1/31 (3) | 12/62 (19) |
| May 2021 and onwards | 28/31 (90) | 34/62 (55) |
| Focal neurologic deficits at presentation | 11/31 (35) | 24/62 (39) |
| Coma at presentation | 3/30 (10) | 1/61 (2) |
| Seizure at presentation | 13/30 (43) | 19/62 (31) |
| Concomitant VTE at presentation | 2/29 (7) | 2/61 (3) |
| Intracranial hemorrhagic lesion | 13/31 (42) | 17/62 (27) |
|  |  |  |
| **Laboratory data, n/N (%)** |  |  |
| Thrombocytopenia at any time during admission | 2/30 (7) | 4/62 (6) |
| Platelet count nadir, median (IQR), x10^3^/µL^e^ | 250 (222-282)^f^ | 247 (195-307) |
| Anti-PF4 antibodies |  |  |
| Positive | 0/31 | 2/62 (3) |
| Negative | 10/31 (32) | 18/62 (29) |
| Not tested or unknown | 21/31 (68) | 42/62 (68) |
| D-dimer level (highest value)^g^ |  |  |
| >4 µg/mL FEU | 1/31 (3) | 0/62 |
| 2-4 µg/mL FEU | 2/31 (6) | 4/62 (6) |
| <2 µg/mL FEU | 11/31 (35) | 39/62 (63) |
| Not tested or unknown | 17/31 (55) | 19/62 (31) |

CVST = cerebral venous sinus thrombosis; FEU = fibrinogen equivalent units; IQR = interquartile range; PF4 = platelet factor 4; VITT = vaccine-induced immune thrombotic thrombocytopenia; VTE = venous thromboembolism.

^a^Six cases after Covishield (Serum Institute of India) vaccination and five cases after Vaxzevria (Oxford/AstraZeneca) vaccination; ^b^All Vaxzevria (Oxford/AstraZeneca); ^c^Within 12 weeks; ^d^In last 10 years; ^e^To convert to 10^9^/L, multiply values by 1; ^f^Two missing values; ^g^To convert to nmol/L, multiply values by 5·476.

**Supplemental Table 6. Treatment and outcomes of CVST cases with unlikely VITT from middle- and high-income countries.**

|  | **Unlikely CVST-VITT cases from middle-income countries**  **(N = 31)** | **Unlikely CVST-VITT cases from high-income countries**  **(N = 62)** |
| --- | --- | --- |
| **Treatment data, n/N (%)** |  |  |
| Any anticoagulant treatment | 29/31 (94) | 62/62 (100) |
| Non-heparin as first anticoagulant^a^ | 3/29 (10) | 9/62 (15) |
| Endovascular treatment | 2/31 (6) | 3/62 (5) |
| Decompressive neurosurgery | 1/31 (3) | 1/62 (2) |
| Intensive care unit admission | 6/31 (19) | 16/62 (26) |
|  |  |  |
| **Clinical events during admission, n/N (%)** |  |  |
| New concomitant VTE | 1/31 (3) | 0/60 |
| Bleeding complication | 2/31 (6) | 4/60 (7) |
| Major bleeding^b^ | 2/31 (6) | 4/60 (7) |
|  |  |  |
| **Discharge data, n/N (%)** |  |  |
| Duration hospital admission, median (IQR), days | 7 (4-12) | 7 (3-12) |
| Discharge disposition |  |  |
| Home | 28/30 (93) | 45/62 (73) |
| Rehabilitation center | 0/30 | 8/62 (13) |
| Nursing home | 0/30 | 1/62 (2) |
| Other hospital | 0/30 | 4/62 (6) |
| Deceased | 2/30 (7) | 4/62 (6) |
|  |  |  |
| **Follow-up, n/N (%)** |  |  |
| Time from diagnosis to latest follow-up, median (IQR), days | 113 (24-176) | 108 (49-170) |
| mRS score at latest follow-up |  |  |
| mRS 0 | 14/31 (45) | 24/62 (39) |
| mRS 1 | 10/31 (32) | 22/62 (35) |
| mRS 2 | 4/31 (13) | 7/62 (11) |
| mRS 3 | 1/31 (3) | 2/62 (3) |
| mRS 4 | 0/31 | 3/62 (5) |
| mRS 5 | 0/31 | 0/62 |
| mRS 6 | 2/31 (6) | 4/62 (6) |

CVST = cerebral venous sinus thrombosis; IQR = interquartile range; mRS = modified Rankin Scale; VITT = vaccine-induced immune thrombotic thrombocytopenia; VTE = venous thromboembolism.

^a^No low‑molecular‑weight heparin or unfractionated heparin; ^b^According to the criteria of the International Society on Thrombosis and Haemostasis.^12^

**Supplemental Figure 1. Flowchart of patient selection.**

**
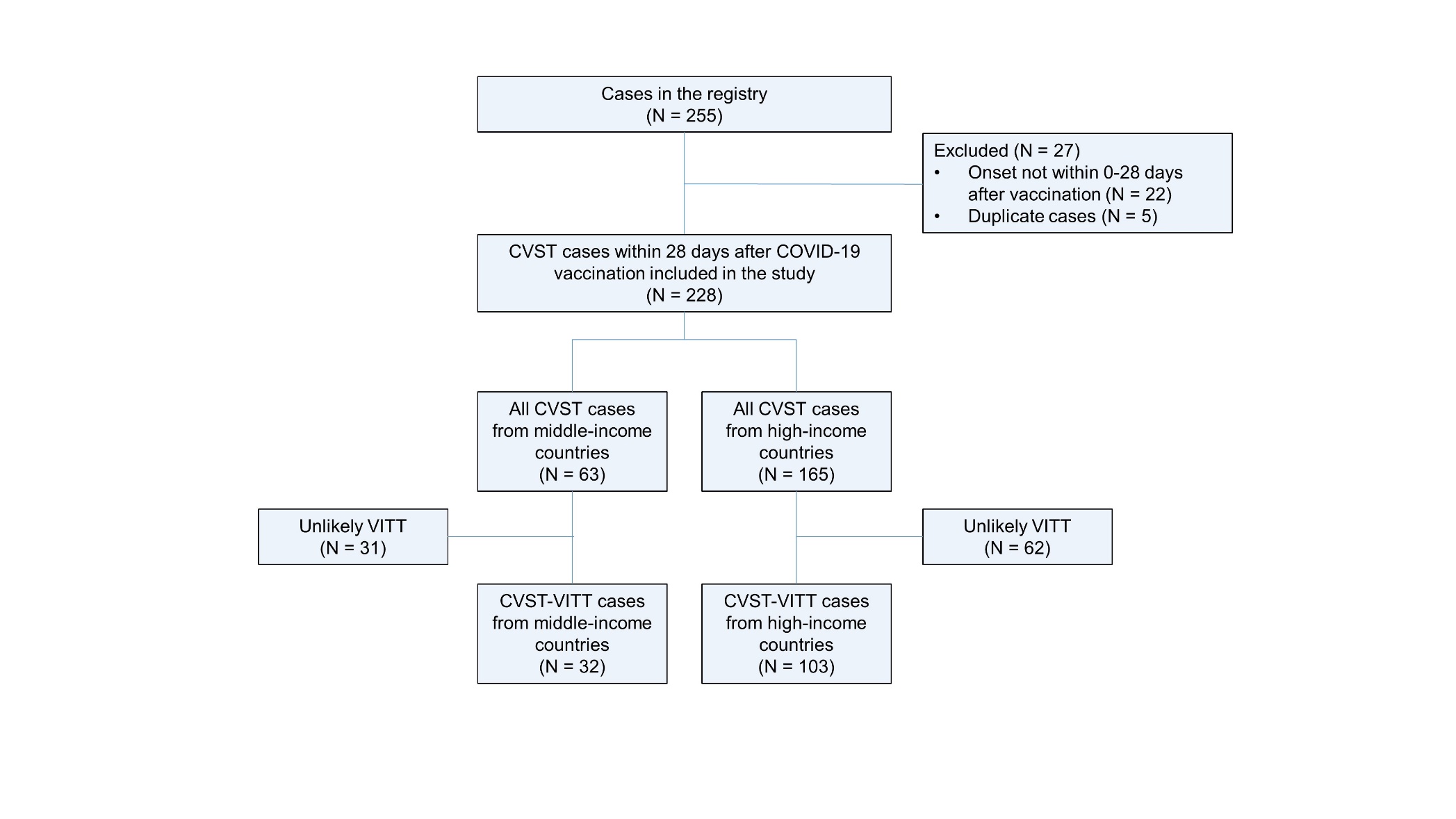
**

CVST=cerebral venous sinus thrombosis; VITT=vaccine-induced immune thrombotic thrombocytopenia.

**Supplemental Figure 2. mRS score at latest follow-up of CVST-VITT patients from middle-income countries and high-income countries.**

**
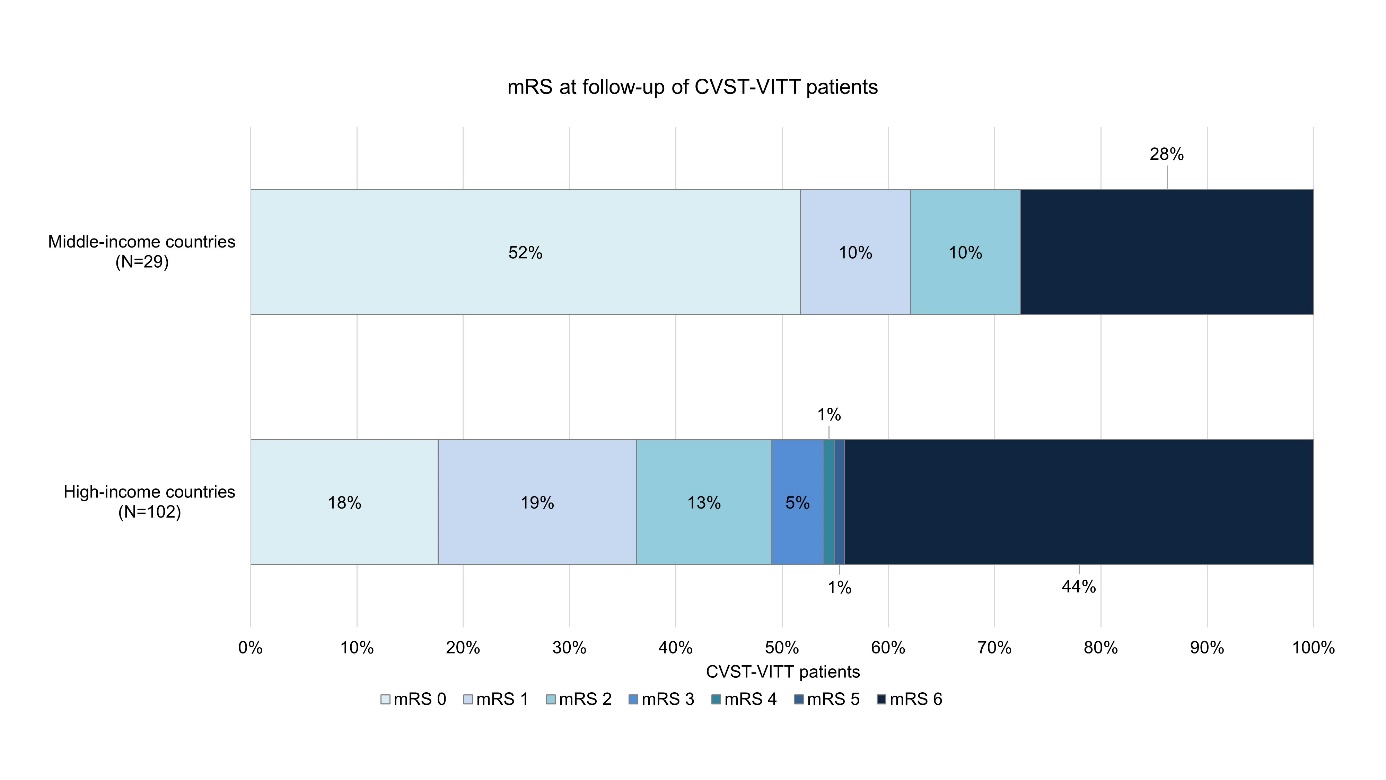
**

CVST=cerebral venous sinus thrombosis; mRS=modified Rankin Scale; VITT=vaccine-induced immune thrombotic thrombocytopenia. The median time from diagnosis to latest follow-up was 33 (IQR 10-97) days in middle-income countries and 59 (IQR 3-151) days in high-income countries. There were three missing values in the middle-income country group and one missing value in the high-income country group.

**Supplemental References**

1. Pavord S, Scully M, Hunt BJ, et al. Clinical Features of Vaccine-Induced Immune Thrombocytopenia and Thrombosis. *N Engl J Med* 2021; 385: 1680-1689. 2021/08/12. DOI: 10.1056/NEJMoa2109908.

2. The World Bank Group. World Bank Open Data, <https://data.worldbank.org/> (accessed May 15 2023).

3. Ministério da Saúde. Vacinômetro COVID-19, <https://infoms.saude.gov.br/extensions/SEIDIGI_DEMAS_Vacina_C19/SEIDIGI_DEMAS_Vacina_C19.html> (accessed May 15 2023).

4. Xuanwu Hospital of Capital Medical University. Introduction, <https://www.xwhosp.com.cn/Html/News/Articles/10008745.html> (accessed May 12 2023).

5. Xuanwu Hospital of Capital Medical University. Neurology, <https://www.xwhosp.com.cn/Html/News/Articles/10008727.html> (accessed May 12 2023).

6. CoWIN. CoWIN Dashboard, <https://dashboard.cowin.gov.in/> (2021, accessed August 23 2022).

7. Mathieu E, Ritchie H, Ortiz-Ospina E, et al. A global database of COVID-19 vaccinations. *Nat Hum Behav* 2021; 5: 947-953. 20210510. DOI: 10.1038/s41562-021-01122-8.

8. Shiraz University of Medical Sciences. <https://sib.sums.ac.ir/> (accessed May 10 2023).

9. INNN. El Instituto, <http://www.innn.salud.gob.mx/interna/instituto/instituto.html> (accessed May 15 2023).

10. Reuters. COVID-19 Tracker Pakistan, <https://www.reuters.com/graphics/world-coronavirus-tracker-and-maps/countries-and-territories/pakistan/> (accessed May 15 2023).

11. van de Munckhof A, Lindgren E, Kleinig TJ, et al. Outcomes of Cerebral Venous Thrombosis due to Vaccine-Induced Immune Thrombotic Thrombocytopenia After the Acute Phase. *Stroke* 2022: 101161STROKEAHA122039575. 2022/09/10. DOI: 10.1161/STROKEAHA.122.039575.

12. Schulman S, Kearon C, Subcommittee on Control of Anticoagulation of the S, et al. Definition of major bleeding in clinical investigations of antihemostatic medicinal products in non-surgical patients. *J Thromb Haemost* 2005; 3: 692-694. 2005/04/22. DOI: 10.1111/j.1538-7836.2005.01204.x.
